# KRT17 from skin cells with high glucose stimulation promotes keratinocytes proliferation and migration

**DOI:** 10.3389/fendo.2023.1237048

**Published:** 2023-10-19

**Authors:** Peng Zhou, Haijun Feng, Wenhui Qin, Qin Li

**Affiliations:** ^1^ Department of Vascular Surgery, Union Hospital, Tongji Medical College, Huazhong University of Science and Technology, Wuhan, China; ^2^ Department of Vascular Surgery, The Central Hospital of Wuhan, Tongji Medical College, Huazhong University of Science and Technology, Wuhan, China; ^3^ Department of Endocrinology, Jingshan Union Hospital of Huazhong University of Science and Technology, Jingshan, China

**Keywords:** diabetic wound healing, HaCaT cells, Krt17, proliferation, migration

## Abstract

Impaired diabetic wound healing is an important issue in diabetic complications. Proliferation and migration of keratinocytes are major processes of skin wound repair after injury. However, hyperkeratosis can affect the speed of wound healing. Based on the results of preliminary experiments on increased KRT17 expression after high glucose stimulation of human skin tissue cells, a cell model of human immortalized keratinocyte (HaCaT) stimulation with different concentrations of KRT17 was established *in vitro*, and the promotion in cell proliferation and migration were discovered. KRT17 silencing promoted diabetic wound healing in the db/db diabetic wound model. Transcriptome sequencing (RNA-seq) was performed on HaCaT cells after KRT17 stimulation, and analysis showed significant enrichment in the PI3K-AKT signaling pathway, in which the regulation of cell c-MYB mRNA, a key molecule regulating cell proliferation and migration, was significantly upregulated. *In vitro* assays showed increased c-MYB expression and enhanced pAKT activity after HaCaT cell stimulation by KRT17. We speculate that KRT17 is upregulated under high glucose and promotes keratinocyte proliferation and migration caused hyperkeratosis, through the c-MYB/PI3K-AKT pathway, contributing to delayed wound healing.

## 1 Background

Skin complications are common in diabetic patients, with reports suggesting that 30%-91% of diabetic patients experience at least one diabetic complication in their lifetime (1). Common skin lesions include infections, pruritus, erythema, sclerosing edema, lipid-like progressive necrosis, and delayed wound healing. Researchers have made vast strides in recent years, with the discovery that the skin structure of diabetic patients is altered early in the course of diabetes and sustained disruption of the skin structure may lead to a range of skin lesions (2). The specific mechanisms underlying the different skin lesions in diabetes remain unclear, and their severity, incidence and response to treatment are highly heterogeneous. One of the most serious skin complications is delayed healing of diabetic wounds, especially in the foot, which can lead to chronic ulcers and eventually to the diabetic foot, a serious threat to limb safety.

To study the changes in the skin under diabetic pathological conditions, we established models using three major cells in human skin tissue stimulated by high glucose *in vitro*, including Human Epidermal Keratinocytes (HEK), Human Dermal Fibroblasts (HDF) and Human Dermal Microvascular Endothelial Cells (HDMEC). RNA-seq analysis of the three cells revealed that 16 differential genes were co-expressed in the three cells under high glucose stimulation, of which only keratin 17 (KRT17) mRNA was consistently upregulated in all three cells suggesting that KRT17 may play an important role in diabetic skin lesions (3).

In the skin, keratin is the main structural component of the epidermis, nails and hair, and in the fully differentiated epidermis, keratin accounts for up to 85% of the total cellular proteins, mainly forming the cytoskeleton and maintaining the structural integrity of the cell (4). Keratin can be classified into types I and II based on genetic isoforms and protein sequence homology of the central α-helical rod-like structural domain, which are present in pairs in epithelial cells (5). KRT17 is an important component of the type I KRT family and is a highly conserved protein. It has been established that KRT17 is not expressed in normal skin but is induced in large amounts in skin stress states, such as skin injury, viral infections and psoriasis (6–8). Interestingly, KRT17 is a multifunctional regulatory protein capable of regulating various cellular biological processes, including cell growth and proliferation (9, 10), skin inflammation, skin adnexa differentiation (11, 12), and epithelial tumor or epithelioid tumorigenesis and invasion (13, 14).

The skin consists of the epidermis, dermis and subcutaneous tissue. The epidermis is the outermost layer of the skin, and keratin-forming cells are its main component. According to the different developmental stages and morphological characteristics of keratin-forming cells, it can be divided into 5 layers: stratum corneum, stratum hyaline, stratum granulosum, stratum spinosum, and stratum basale (15). Keratin-forming cells are considered to be an important component in maintaining epidermal homeostasis and promoting epidermal renewal. In addition, keratin-forming cells play an important role in the immune defense process of the skin. During wound repair, keratin-forming cells are involved in the reepithelialization process through proliferation, migration and differentiation, as well as in wound contraction together with fibroblasts to restore epidermal integrity. In addition, keratin-forming cells can interact with immune cells through various cytokines to accurately coordinate the activities of different cells and ensure the smooth progress of wound repair (16, 17).

Despite the deluge of studies about KRT17 being carried out in skin appendage development, psoriasis, wound healing and oncology, the relevant studies in diabetic wound healing are scarce. To clarify the effect of KRT17 on the skin, we first designed an *in vitro* stimulation of skin keratin-forming cells by KRT17 to explore its effect on epidermal function. We observed the effect of KRT17 on skin keratin-forming cell proliferation and migration and then clarified the effect of KRT17 with increased expression under high glucose on the skin to explore the possible mechanism of diabetic skin lesion occurrence and development.

## 2 Methods

### 2.1 Cell culture

The human immortalized keratinocyte (HaCaT) cell line was cultured in MEM (Cat #PYG0029, Boster) medium with 10% FBS (Cat # 10099141C, GIBCO) and 1% penicillin/streptomycin at 37°C in a 5% CO_2_ humidified incubator. The HaCaT cell lines present in this study were obtained from Procell Life Science&Technology Co.,Ltd, Cat #CL-0090 (Wuhan, China).

### 2.2 Stimulation of recombinant human cytokeratin 17

HaCaT cells were seeded in 6-well plates and cultured. At 60%-70% confluence, HaCaT cells were starved in serum-free MEM for 12h before stimulation with 0.1, 1 and 10ng/ml recombinant human cytokeratin 17 (KRT17, Cat#PRO-1883, ProSpec).

### 2.3 Observation of cell morphology and growth

The growth of the cells stimulated with different concentrations of KRT17 was observed and recorded under an inverted microscope daily.

### 2.4 Cell proliferation assay

Cell proliferation was assessed using Cell Counting Kit-8 (CCK8, Cat #CK04-500T, Dojindo). HaCaT cells were seeded in 96-well plates at a density of 1x10^4^ cells/well a day before the replacement of fresh media containing different concentrations of KRT17 at 0, 0.1, 1 and 10 ng/ml. After 24h, 48h and 72h, cell proliferation was measured by the CCK-8 assay. The experimental procedures were performed as described by Shidi Wu (18), one of our Lab Members. Briefly, when performing the CCK-8 assay, 100μl fresh media containing 10μl CCK-8 was added to each well, and then the plate was incubated for 1h at 37°C away from light. Absorbance was measured at a wavelength of 450 nm by a Thermo Scientific Microplate Reader (Thermo Fisher, USA).

### 2.5 Cell migration assay

Cell migration was assessed by scratch and Transwell assays. HaCaT cells were seeded in three 12-wells plates for each treatment. When the cell confluency reached 90%-95%, three different scratches were made in each well. A scratch was made using the 20μl pipette tip along the diameter of the well. Then, cells were starved in a serum-free culture medium for 24 h. Cells were washed with PBS to remove the scratched cells. Fresh complete culture media was added containing different concentrations of KRT17 (0 and 1 ng/mL KRT17). Afterward, images were taken from the same area for each treatment condition at 0, 12 and 24 h. For the Transwell assay, 24-well Transwell chambers (Corning) were used. HaCaT cells were seeded into the upper layer in basal medium without FBS at a density of 1x10^5^ cells/well, while the lower chamber was filled with different concentrations of KRT17 MEM containing 10% FBS. The invaded cells were fixed and quantified after 24 hours.

### 2.6 RNA extraction and RNA sequencing procedures

RNAs were extracted from cultivated HaCaT cells using RNAiso Plus (Cat # 9108, TaKaRa). RNA sequencing (RNA-seq) and RNA-seq analysis were performed on a commercially available service (service ID # F21FTSCCWGT0114, BGI, Wuhan, China). The procedures were performed as previously described (3). Briefly, total RNA was extracted, and mRNA was enriched by Oligo (dT) bead for library construction. After the library was constructed, the qualified library was chosen for sequencing. Following the sequencing of each cDNA library, the raw sequencing data were transformed into the original sequence data termed raw data or raw reads. Raw sequencing reads QC and filtering were done with Fastp. After filtering, the clean reads were aligned against the reference genome. The clean reads were processed with downstream analysis, including gene expression and deep analysis based on gene expression.

### 2.7 Animal and experimental design

We purchased db/db mice (Cat# HM0046, shulb) to establish a diabetic wound healing animal model. A round full-thickness skin defect wound model with a diameter of 0.6 cm was cut on both sides of the back vertebrae. The left and right sides administration of NC siRNA and KRT17 siRNA (Cat #sc-43312, Santa) emulsions mixtures (Lipofectamine3000), respectively. On the 0th, 3rd, 6th, 9th, 12th days after wound manufacturing, the wound healing situation was recorded. The project was approved by the Wuhan Union Hospital Ethics Committee (NO. 3110).

### 2.8 Functional enrichment analysis

Normalization and differential expression analyses were estimated from count data using DEGseq package in analysis system of Dr. Tom from BGI. Differentially expressed genes (DEGs) were screened using the criteria: FDR-adjusted p values (p values ≤ 0.05) and fold changes ≥1.2. DEGs were extracted for GO functional enrichment analysis using the GO online analysis tool DAVID (https://david.ncifcrf.gov/) and KEGG pathway enrichment analysis using KEGG online tools (http://www.kegg.jp/). Data visualization was done in GraphPad Prism9.

### 2.9 Quantitative real-time RT-PCR

The experiments were performed as previously described (19). The extracted RNA was reverse-transcribed into cDNA using a cDNA synthesis kit (Cat # RR037A, Takara). Real-time quantitative PCR was implemented on an ABI StepOne Plus System (Applied Biosystems, Foster City, CA) using SYBR Premix Ex Taq (Cat # RR420A, Takara). The primers used were c-MYB, 5’- GAAAGCGTCACTTGGGGAAAA-3’ (forward) and 5’- TGTTCGATTCGGGAGATAATTGG-3’ (reverse). All primers were purchased from Sangon Biotech. The mRNA levels of the target genes were normalized to GAPDH using the 2−DDCT method.

### 2.10 Immunofluorescence staining

The experimental procedures were performed as described by Wu (18). HaCaT cells were seeded on circular coverslip slides in 24-well plates at a density of 5000 cells/well and cultured. After treatment with 1ng/ml KRT17 complete medium for 24h, HaCaT cells were washed twice with PBS, fixed with 4% paraformaldehyde for 30 minutes and rinsed twice with PBS. Then cells were treated with 0.1% Triton-X 100 for 10 minutes. After blocking the slides with 5% goat serum (Boster, Wuhan, China) for 1 hour, c-MYB primary antibodies (1:50, Cat #17800-1-AP, Proteintech) were incubated overnight at 4°C. Secondary antibodies labeled by FITC (Green) (1:200, Servicebio) were incubated with HaCaT cells at 37°C for 1h in the dark. Cells were subsequently stained with DAPI (Blue) for 5min away from light. Images were acquired on a fluorescence microscope (BIO-RAD).

### 2.11 Western blotting

Total proteins from the HaCat cells in 6-well plates were extracted using RIPA lysis buffer with 2% PMSF and phosphatase inhibitor (Cat # P0013K, Beyotime). The experiments were performed as previously described (20). The extracted proteins were separated using 10%SDS electrophoresis before transfer onto a nitrocellulose membrane. The membrane was separately probed by incubation with the respective primary antibody (c-MYB, Cat #17800-1-AP, Proteintech) overnight at 4°C, followed by incubation with horseradish peroxidase-labeled secondary antibody for 2 h at 37°C. Enhanced chemiluminescence reagent (Cat # MA0186, Meilunbio) was then added to the blots, and the bands were analyzed using ImageJ software (NIH, USA).

### 2.12 Statistical analyses

Data were expressed as the mean ± SEM. Parametric and non-parametric quantitative variables were compared using the Student’s t-test and the Mann–Whitney U test, respectively. The least significant difference (LSD) method in one-way ANOVA was used for pairwise comparisons between different groups. P-values < 0.05 were statistically significant. All figures were generated using GraphPad Prism 9.0 and Adobe Illustrator CC 2015.

## 3 Results

### 3.1 KRT17 promotes HaCaT cell proliferation

**Figure 1:**
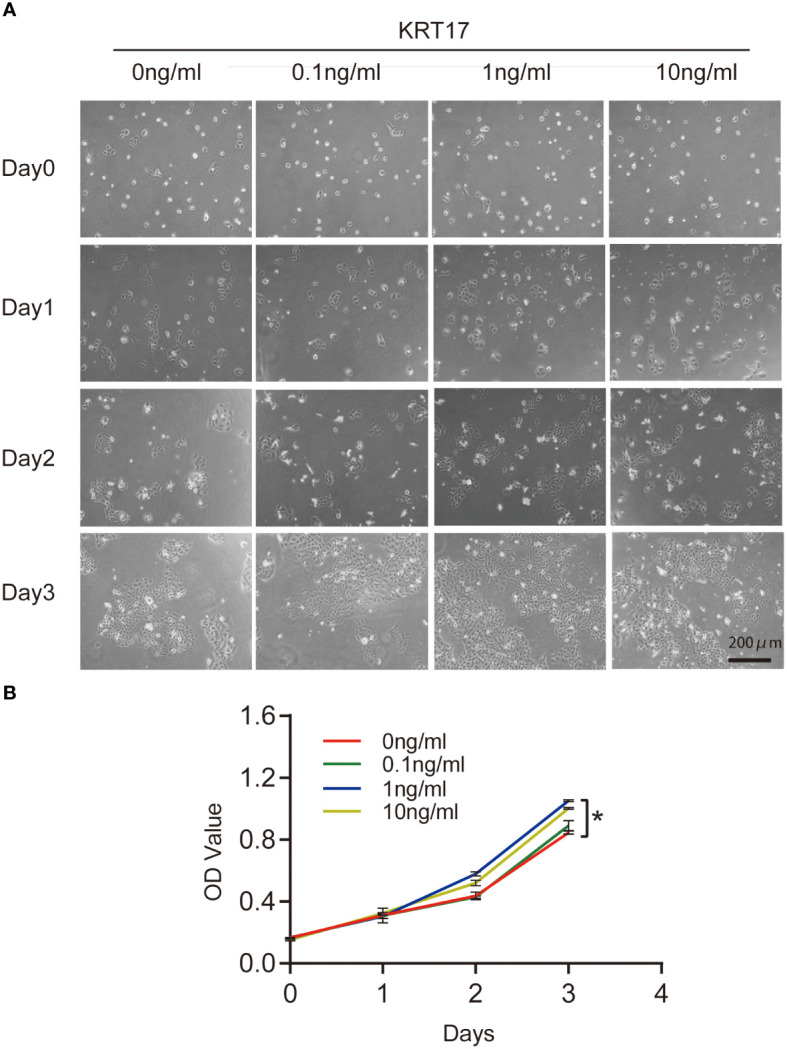
KRT17 promote HaCaT cell growth and proliferation. **(A)** Cell morphological changes and high-density growth under a microscope. **(B)** A graph of the CCK8 assay results. Maximal promote was seen in the presence of 1 ng/ml of KRT17 (Blue) versus the control (Red). The independent experiment was repeated three times. The results are provided as the means ± SEM, *p < 0.05 compared with the control. All studies were performed in triplicate and independently repeated three times.

To investigate the effect of KRT17 on HaCaT proliferation, the culture was stimulated by 0.1, 1 and 10ng/ml KRT17. The microscopic observation indicated that the growth of HaCaT cells in the control and 0.1ng/ml KRT17 stimulation groups was not significantly different, while cell proliferation and clustering in the 1 and 10ng/ml KRT17 stimulation groups grew faster (**Figure 1A**). The CCK8 assay revealed that stimulation with KRT17 could promote HaCaT cell proliferation (**Figure 1B**). The most significant proliferative ability was observed with 1 ng/ml KRT17 (blue curve). Therefore, we selected a KRT17 concentration of 1 ng/ml for the subsequent experiments.

### 3.2 KRT17 promotes HaCaT cell migration

**Figure 2:**
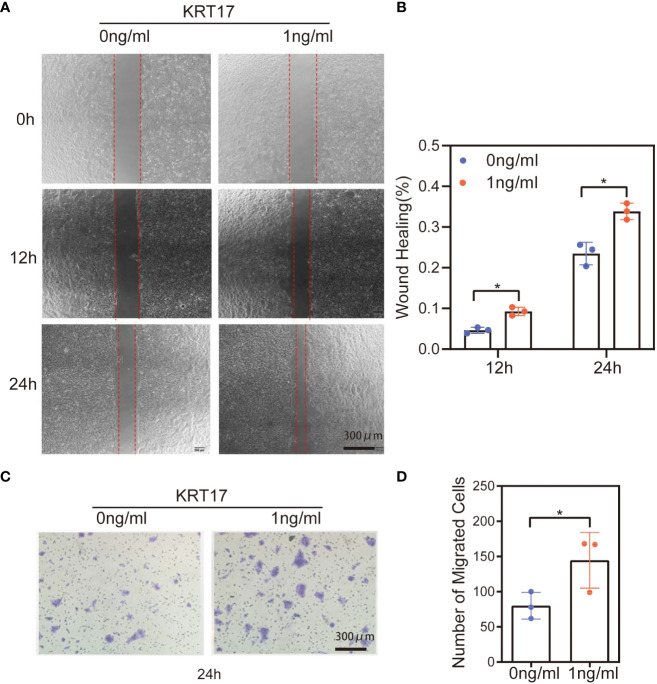
KRT17 increases HaCaT cell migration. **(A)** Results of cell scratching wound healing assay. **(B)** Statistical analysis of cell migration in the scratch wound healing assays. **(C)** Transwell invasion assay results. **(D)** Statistical analysis of the results from the Transwell migration assays. The independent experiment was repeated three times. The results are provided as the means ± SEM, *p < 0.05 compared with the control. All studies were performed in triplicate and independently repeated three times.

HaCaT cell migration in response to KRT17 was measured using scratch wound assays and Transwell migration assays. The scratch wound assay showed that KRT17 promoted HaCaT cell migration at different time intervals (12h & 24h) (**Figure 2A**). The statistical results are displayed in **Figure 2B**. Similarly, the results of the Transwell assay demonstrated that KRT17 stimulation significantly promoted the migration ability of HaCaT cell (**Figure 2C**). The statistical results are displayed in **Figure 2D**.

### 3.3 KRT17 inhibition accelerated wound healing of db/db diabetic mice

**Figure 3:**
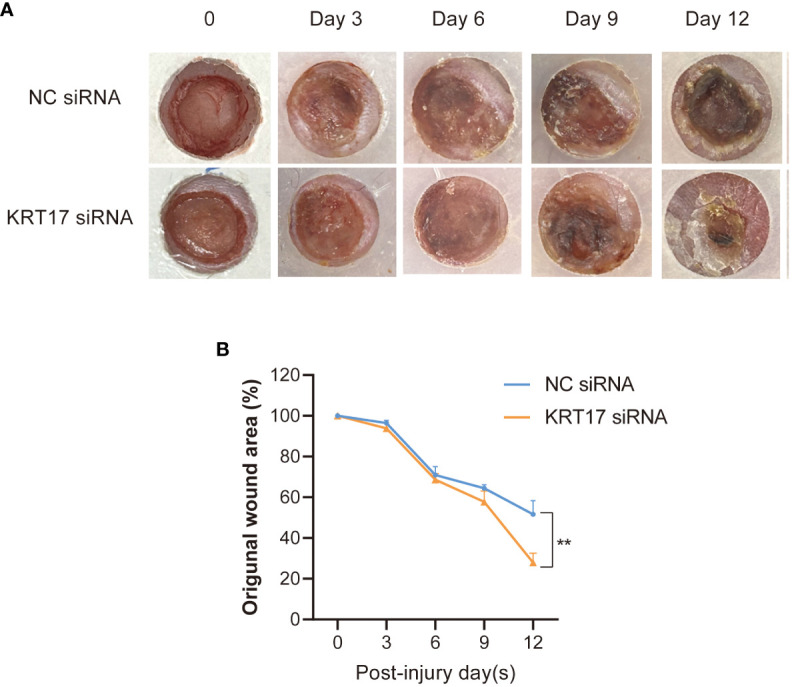
KRT17 silencing promoted diabetic wound healing. Images **(A)** and quantification **(B)** of wound area in KRT17 siRNA emulsions mixtures -treated group and control group. **p<0.01.

To further elucidate the effect of KRT17 on diabetic wound healing *in vivo*, the effect of KRT17 siRNA emulsions mixtures on db/db diabetic mice wound model was employed. Images of the wounds were captured every 3 days, and a significantly higher healing rate was observed in the KRT17 siRNA emulsions mixtures -treated group (**Figures 3A, B**).

### 3.4 GO analysis of differentially expressed genes

**Figure 4:**
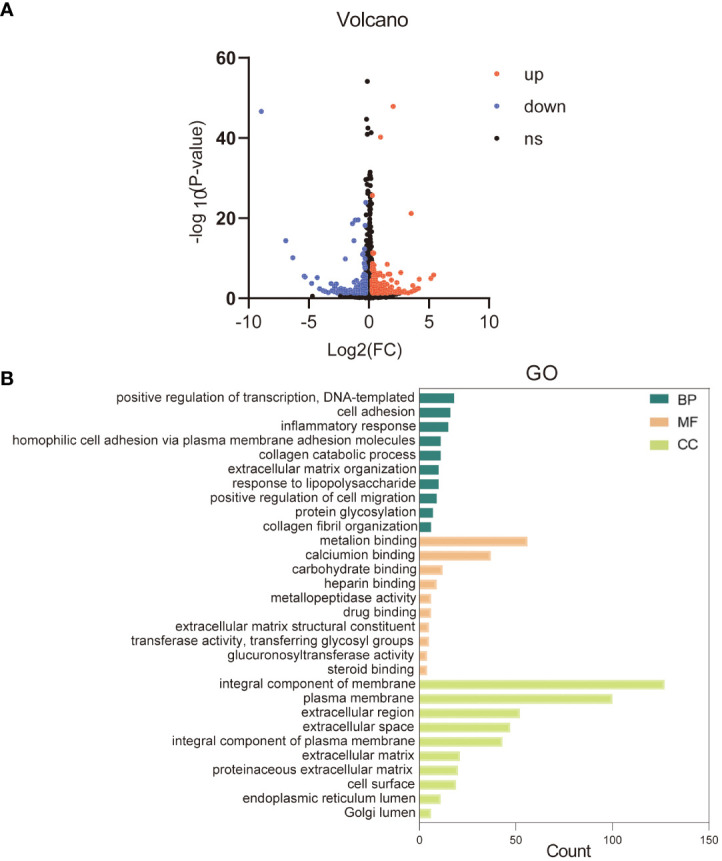
Gene ontology (GO) and enrichment analysis of the differentially expressed genes. **(A)** The volcano plot of differentially expressed genes. **(B)** Top 10 GO categories are shown. Green represents the top 10 GO terms of biological process (BP), orange represents the top 10 GO terms of molecular function (MF), and yellow represents the top 10 GO terms of cellular component (CC).

To better understand the cellular response of HaCaT to KRT17, we performed RNA sequencing (RNA-seq) of KRT17-exposed cells. 493 DEGs were screened from common 15563 genes, including 196 downregulated and 297 upregulated genes. The data are visualized in a volcano plot (**Figure 4A**). GO analysis of DEGs was conducted according to three GO categories, biological process (BP), molecular functions (MF) and cellular component (CC). The top 10 enriched GO terms in each GO category are displayed in **Figure 4B**. Significantly enriched GO terms for biological processes included positive regulation of transcription, cell adhesion, inflammatory response, etc. In terms of molecular functions, metal ion binding, calcium ion binding, and carbohydrate binding were significantly enriched. Finally, for the cellular component category, integral component of membrane, plasma membrane, and extracellular region were significantly enriched.

### 3.5 The PI3K-AKT signaling pathway is significantly activated during KEGG pathway analysis

**Figure 5:**
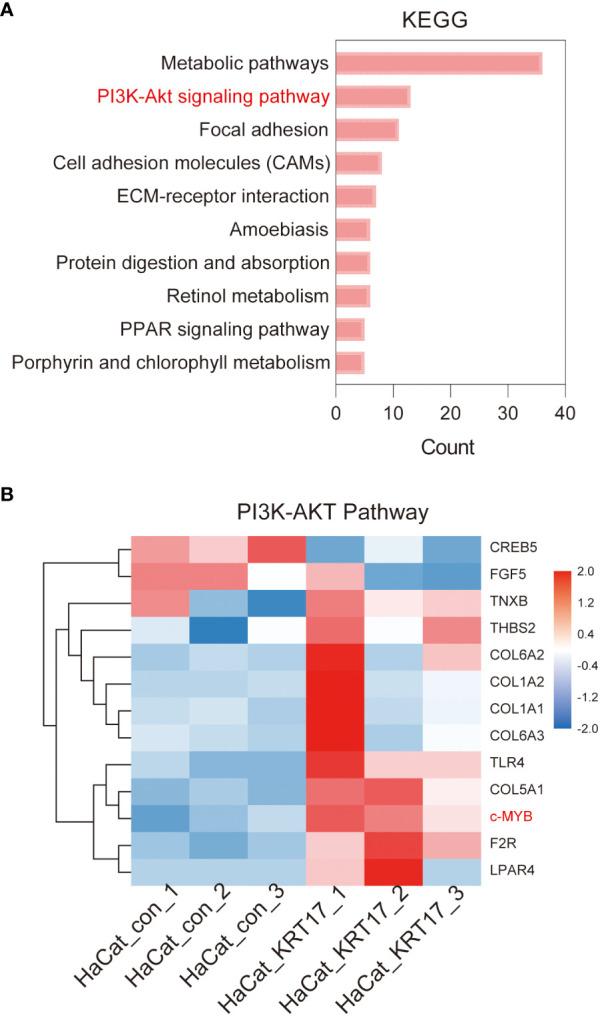
KEGG enrichment analysis of differentially expressed genes. **(A)** Top 10 KEGG pathways of KEGG enrichment analyses of differentially expressed genes. **(B)** The heatmap of the differential gene expression analysis based on the enriched PI3K/AKT signaling pathway. The upregulated gene c-MYB that suggested involved in cell proliferation and migration is marked (red).

KEGG enrichment analysis of DEGs showed that the DEGs of KRT17 stimulation were mainly enriched in metabolic pathways, PI3K-Akt signaling pathway, Focal adhesion, etc. The top 10 significant KEGG pathways ranked by gene count are shown in **Figure 5A**. As the PI3K-AKT pathway is a crucial signaling pathway in cellular processes such as proliferation and migration, the thirteen target genes in the PI3K/AKT pathway were analyzed in detail (**Figure 5B**). Eleven of these 13 genes were upregulated and 2 were downregulated. Of these upregulated genes, c-MYB is a transcription factor involved in cell cycle progression, cell proliferation and differentiation, suggesting involvement in cell proliferation and migration after KRT17 stimulation.

### 3.6 Akt activity and c-MYB expression were elevated in HaCaT cells after KRT17 stimulation

**Figure 6:**
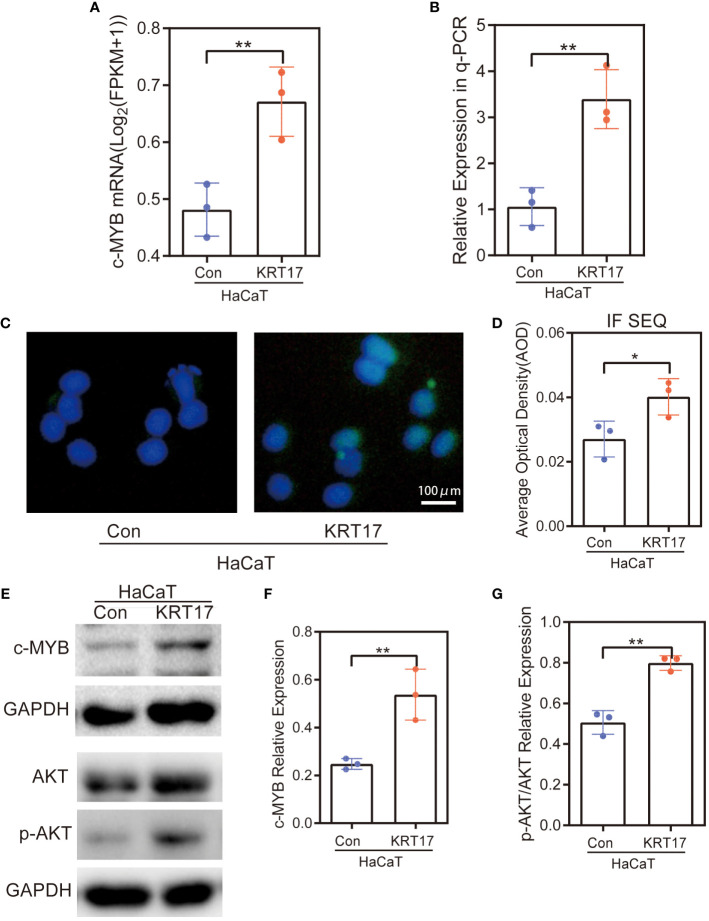
c-MYB expression was elevated and PI3K/AKT signaling pathway was activated in HaCaT cells after KRT17 stimulation. **(A)** RNA-seq quantification of the expression of c-MYB mRNA. **(B)** RT‐qPCR validation of the expression of c-MYB. **(C, D)** c-MYB Immunofluorescence and quantitative analysis. **(E-G)** Western blots and quantitative analysis of c-MYB and p-AKT. The independent experiment was repeated three times. The results are provided as the means ± SEM, *p < 0.05 compared with the control. All studies were performed in triplicate and independently repeated three times. **p<0.01.

We next performed experiments to verify whether KRT17 stimulation increased the expression of related target genes. During RNA-seq, the increased c-MYB mRNA expression levels were observed in HaCaT cells with KRT17 stimulation (**Figure 6A**). Consistent with RNA-seq results, qPCR results showed c-MYB expression was increased (**Figure 6B**). To further validate the above results, immunofluorescence staining was performed, showing increased c-MYB protein expression levels (**Figure 6C**). Quantification of the fluorescence intensity was subsequently performed (**Figure 6D**). Western Blot analysis showed increased c-MYB and p-AKT expression (**Figure 6E**). Finally, quantification of protein levels was performed by western blot (**Figures 6F, G**).

## 4 Discussion

To investigate the gene expression changes and interactions of skin cells under high glucose, our group established three major skin cell models (HEK, HDF and HDMEC) stimulated by high glucose and performed RNA-seq analysis on the cell samples. We found that 16 DEGs were co-expressed in the three cells under high glucose stimulation, among which only KRT17 was expressed in all three cells with consistent changes and upregulated expression. This finding indicates that KRT17 may play an important role in diabetic skin lesions. To clarify the effect of KRT17 on skin keratin-forming cells, we established a cell model of KRT17-stimulated HaCaT *in vitro*; we observed the changes in proliferation and migration of HaCaT cells and performed RNA-seq analysis. The results showed that KRT17 promotes keratinocyte proliferation and migration through the c-MYB/PI3K-AKT pathway. We hypothesize that KRT17, which is upregulated under diabetic pathological conditions, could play a regulatory role in diabetic skin lesions through its effects on HaCaT cell proliferation and migration.

Over the years, studies have demonstrated that KRT17 is a backbone protein capable of performing many biological functions while maintaining the structural stability of cells. It was initially found that Pachyonychia Congenita are due to KRT17 gene defects (21, 22). Subsequently, studies demonstrated that KRT17 is a stress molecule that plays a regulatory function when the skin is exposed to external stimuli (23), playing an immunomodulatory function in the development of psoriasis (24). An increasing body of evidence from recently published studies found that KRT17 plays a key role in promoting epithelial tumorigenesis and metastasis (13, 14). Indeed, much emphasis has been placed on better understanding the biological functions of KRT17 to provide new therapeutic targets for treatment. A recent study by our research team found that *in vitro* KRT17 stimulation promotes the proliferation and migration of skin keratin-forming cells, suggesting that KRT17 may play an important role in diabetic skin complications, especially delayed wound healing.

Significant enrichment analysis of RNA-seq differentially expressed gene KEGG Pathway after KRT17 stimulation of HaCaT showed that the signaling pathway enriched to a higher number of genes was PI3K-AKT signaling pathway. Overwhelming evidence substantiates that the PI3K-AKT signaling pathway is important in regulating cell proliferation and migration (25–27). Accordingly, we screened 13 differential genes in the PI3K-AKT signaling pathway and found that c-MYB, a key molecule capable of regulating cell proliferation and migration, was significantly upregulated. The upregulation of c-MYB and activation of the PI3K-AKT signaling pathway were subsequently validated at the molecular, protein and cellular levels in a KRT17-stimulated HaCaT cell model.

It is widely acknowledged that the PI3K-AKT pathway is a classical signaling pathway that regulates cell proliferation and migration and can be activated by various regulatory proteins. c-MYB is a highly conserved transcription factor that forms the MYB family with A-MYB and B-MYB. MYB proteins are differentially expressed in different tissues and exert diverse biological functions in combination with other co-factors (28). Moreover, MYB family members are frequently aberrantly expressed in human cancers, suggesting that they may be important for tumor initiation and/or maintenance (29). Interestingly, the aberrant expression of c-MYB was first identified in leukemia cells. However, little is currently known about the mechanisms by which c-MYB exerts its biological functions (30–32), although numerous studies have shown that c-MYB can exert regulatory effects through the activation of AKT. Moreover, it has been reported that c-MYB can regulate autophagy through activation of the p-AMPK/AKT pathway involved in the development of irreversible damage to the dental pulp due to diabetes (33) and regulate cochlear hair cells from cisplatin-induced damage (34) through the PI3K/AKT pathway. In a study involving mass spectrometry analysis of the secretome of MYB-regulated and controlled pancreatic cancer cell lines, it was found that knockdown of c-MYB can downregulate the signaling pathways associated with glucose metabolism, the PI3K/AKT signaling pathway and oxidative stress response, among other genes (35). Thus, KRT17, which is upregulated under high glucose conditions, can further promote c-MYB expression and thus activate PI3K/AKT signaling pathway.

Wound repair is divided into four overlapping and highly coordinated phases, hemostasis, inflammation, proliferation and remodeling (36, 37). It has been established that reepithelialization, which involves the proliferation and migration of keratin-forming cells, is responsible for the surface reconstruction of skin wounds and is an important process in wound repair. Reepithelialization begins during the proliferative phase after injury and continues until the wound repair remodeling phase (38). The proliferative and remodelling phases of wound healing are closely connected with the production and reorganization of ECM, which is crucial in determining the extent of scarring. One of the hallmarks of the ECM remodeling is the disappearance of ECM collagen, physiologic wound healing requires considerable ECM remodeling and the eventual replacement of the provisional matrix with new collagen fibers. The MMPs are widely known for their roles in tissue remodeling by degrading collagen and other ECM during wound healing (20). After an injury, keratin-forming cells are heavily activated and proliferate, migrate, alter their cytoskeleton, and increase the level of cell surface receptors while generating paracrine signals that coordinate the participation of different surrounding cell types in the repair of injured tissue and responses (39) that are essential for the reepithelialization of the injured area.

There is ample literature substantiating that KRT17 promotes the proliferation and growth of keratinocytes during skin injury and plays an important function in wound reepithelialization (40). Given that oral wound repair is characterized by rapid healing without scar formation, oral wound repair is considered an ideal model for normal wound repair in adult tissues. In a study of oral lichen planus (OLP) and normal skin tissues performing RNA-seq, molecules related to wound healing (41), KRT17, IL36G, TNC and TGFBI genes were significantly upregulated in OLP tissues. Furthermore, it was found that KRT17 expression was downregulated at the edges of unhealed epidermal wounds, along with KRT16, KRT6A and KRT6B, which form heterodimers with each other, play an important role in epidermal regeneration and are epithelial after skin injury overexpressed in normal repair. In addition, keratinocyte migration is deficient in chronic wounds, and keratins KRT16, KRT6a, KRT6b, and KRT17 play an important role in epithelial migration since their downregulation may lead to insufficient epithelial migration (42). Thus, KRT17 is important in skin injury and epithelial repair processes. Considering the remarkable role of KRT17 in promoting skin wound repair, growing evidence suggests that keratin adjuvants accelerate the epithelialization of deep wounds (43, 44) and promote wound healing, providing targets and ideas for treating refractory wounds.

It has been shown that keratin-forming cells are dysregulated in chronic wounds. Failure to re-epithelialize is one of the most obvious signs of chronic wounds (45). The process of wound reepithelialization may occur in various pathological conditions, including diabetes, trauma, burns, and many others (46, 47). Keratosis imperfecta and hyperkeratosis are characteristic of chronic wound keratin-forming cells (48). Hyperkeratosis is primarily associated with chronic wound inflammation, and the microenvironment of hyperinflamed tissue alters the expression of growth factors important for reepithelialization (49, 50). Moreover, hyperkeratosis is detrimental to wound reepithelialization, where keratin-forming cells continue to proliferate and undergo abnormal epidermal differentiation but cannot migrate and close the wound (51).

Although KRT17 is widely believed to promote wound repair and reepithelialization, it remains unclear whether the high expression of KRT17 in trauma tissues leads to excessive epithelial keratinization, which can be detrimental to wound healing. Interestingly, significant upregulation of KRT17 has been found in diabetic foot ulcer tissues; however, keratin subtypes associated with wound activation (KRT6 and KRT16) and with cell differentiation (KRT1, KRT2, and KRT10) were downregulated (52), suggesting that KRT17 upregulation may be involved in delayed healing of diabetic foot ulcers. This finding contrasts with the significant downregulation of KRT17 in chronic non-healing ulcers that impede the healing of chronic ulcers, as mentioned above. Overall, these results suggest that KRT17 may play different functions in regulating wound healing under different pathological conditions, adding to the complexity of the role of KRT17 in regulating wound healing. It remains unknown whether increased KRT17 expression under high glucose that promotes keratinocyte proliferation and migration is a compensatory mechanism of delayed diabetic wound healing or a mechanism leading to the pathogenesis of delayed diabetic wound healing, nor is it clear whether it plays different regulatory roles at different times of wound repair, adding to the complexity of the possible use of KRT17 in clinical treatment, warranting further studies.

Importantly, our study identified that KRT17 promotes skin keratin-forming cell proliferation and migration *in vitro*, and KRT17 silencing promoted diabetic wound healing *in vivo* This study sought to clarify the effects of highly expressed KRT17 on skin cell function under diabetic pathological conditions, and the underlying mechanisms were preliminarily explored. In addition, the effects of KRT17 on other skin cells, such as skin fibroblasts, dermal microvascular endothelial cells, and skin inflammatory cells, which are key directions for our future research.

## Data availability statement

The data presented in the study are deposited in the CNGB Sequence Archive (CNSA) of China National GeneBank DataBase (CNGBdb) with accession number CNP0004789 (https://db.cngb.org/search/project/CNP0004789/).

## Ethics statement

The animal study was approved by Wuhan Union Hospital Ethics Committee [2022], No. 0298. The study was conducted in accordance with the local legislation and institutional requirements.

## Author contributions

QL and HF made substantial contributions to design the research work. PZ and WQ made substantial contributions to complete the study. PZ analysis and interpreted the data, and write the initial draft of the manuscript. QL and HF revised the paper for important intellectual content. All authors have read and approved the final manuscript.

## References

[1] Murphy-Chutorian B Han G Cohen SR. Dermatologic manifestations of diabetes mellitus: A review. Endocrinol Metab Clin North Am (2013) 42(4):869–98. doi: 10.1016/j.ecl.2013.07.004 24286954

[2] Quondamatteo F. Skin and diabetes mellitus: What do we know? Cell Tissue Res (2014) 355(1):1–21. doi: 10.1007/s00441-013-1751-2 24318789

[3] Zhang S Ke Z Yang C Zhou P Jiang H Chen L. High glucose causes distinct expression patterns of primary human skin cells by rna sequencing. Front Endocrinol (2021) 12:603645. doi: 10.3389/fendo.2021.603645 PMC7982678 33763026

[4] Smack DP Korge BP James WD. Keratin and keratinization. J Am Acad Dermatol (1994) 30(1):85–102. doi: 10.1016/S0190-9622(94)70012-5 7506275

[5] Jacob JT Coulombe PA Kwan R Omary MB. Types i and ii keratin intermediate filaments. Cold Spring Harb Perspect Biol (2018) 10(4). doi: 10.1101/cshperspect.a018275 PMC5880164 29610398

[6] Kim S Wong P Coulombe PA. A keratin cytoskeletal protein regulates protein synthesis and epithelial cell growth. Nature (2006) 441(7091):362–5. doi: 10.1038/nature04659 16710422

[7] Proby CM Churchill L Purkis PE Glover MT Sexton CJ Leigh IM. Keratin 17 expression as a marker for epithelial transformation in viral warts. Am J Pathol (1993) 143(6):1667–78. PMC1887274 7504888

[8] Zhang W Dang E Shi X Jin L Feng Z Hu L. The pro-inflammatory cytokine il-22 up-regulates keratin 17 expression in keratinocytes *Via* Stat3 and Erk1/2. PloS One (2012) 7(7):E40797. doi: 10.1371/journal.pone.0040797 22808266 PMC3396590

[9] Depianto D Kerns ML Dlugosz AA Pierre AC. Keratin 17 promotes epithelial proliferation and tumor growth by polarizing the immune response in skin. Nat Genet (2010) 42(10):910–4. doi: 10.1038/ng.665 PMC2947596 20871598

[10] Mikami T Maruyama S Abé T Kobayashi T Yamazaki M Funayama A. Keratin 17 is co-expressed with 14-3-3 sigma in oral carcinoma *In situ* and squamous cell carcinoma and modulates cell proliferation and size but not cell migration. Virchows Archiv Int J Pathol (2015) 466(5):559–69. doi: 10.1007/s00428-015-1735-6 25736868

[11] Chung BM Arutyunov A Ilagan E Yao N Wills KM Coulombe PA. Regulation of c-X-C chemokine gene expression by keratin 17 and hnrnp k in skin tumor keratinocytes. J Cell Biol (2015) 208(5):613–27. doi: 10.1083/jcb.201408026 PMC4347647 25713416

[12] Tong X Coulombe PA. Keratin 17 modulates hair follicle cycling in a tnfalpha-dependent fashion. Genes Dev (2006) 20(10):1353–64. doi: 10.1101/gad.1387406 PMC1472909 16702408

[13] Nair RR Hsu J Jacob JT Pineda CM Hobbs RP Coulombe PA. A role for keratin 17 during dna damage response and tumor initiation. Proc Natl Acad Sci USA (2021) 118(13):e2020150118. doi: 10.1073/pnas.2020150118 33762306 PMC8020757

[14] Quinn JJ Jones MG Okimoto RA Nanjo S Chan MM Yosef N. Single-cell lineages reveal the rates, routes, and drivers of metastasis in cancer xenografts. Sci (New York NY) (2021) 371(6532). doi: 10.1126/science.abc1944 PMC7983364 33479121

[15] Bianchi J Cameron J. Assessment of skin integrity in the elderly 1. Br J Community Nurs (2008) 13(3):S26, S8, S30–2. doi: 10.12968/bjcn.2008.13.Sup1.28684 18557571

[16] Piipponen M Li D Landén NX. The immune functions of keratinocytes in skin wound healing. Int J Mol Sci (2020) 21(22):8790. doi: 10.3390/ijms21228790 33233704 PMC7699912

[17] Suter MM Schulze K Bergman W Monika W Petra R Eliane JM. The keratinocyte in epidermal renewal and defence. Vet Dermatol (2009) 20(5-6):515–32. doi: 10.1111/j.1365-3164.2009.00819.x 20178490

[18] Wu S Zhao M Sun Y Xie M Le K Xu M. The potential of diosgenin in treating psoriasis: Studies from hacat keratinocytes and imiquimod-induced murine model. Life Sci (2020) 241:117115. doi: 10.1016/j.lfs.2019.117115 31790685

[19] Zhou P Guo H Li Y Liu Q Qiao X Lu Y. Monocytes promote pyroptosis of endothelial cells during lung ischemia-reperfusion *Via* il-1r/Nf-κb/Nlrp3 signaling. Life Sci (2021) 276:119402. doi: 10.1016/j.lfs.2021.119402 33785335

[20] Zhou P Yang C Zhang S Ke ZX Chen DX Li YQ. The imbalance of mmp-2/Timp-2 and mmp-9/Timp-1 contributes to collagen deposition disorder in diabetic non-injured skin. Front Endocrinol (2021) 12:734485. doi: 10.3389/fendo.2021.734485 PMC8579102 34777244

[21] Bowden PE Haley JL Kansky A Rothnegal JA Jones DO Turner RJ. Mutation of a type ii keratin gene (K6a) in pachyonychia congenita. Nat Genet (1995) 10(3):363–5. doi: 10.1038/ng0795-363 7545493

[22] Liao H Sayers JM Wilson NJ Irvine AD Mellerio JE Baselga E. A spectrum of mutations in keratins K6a, K16 and K17 causing pachyonychia congenita. J Dermatol Sci (2007) 48(3):199–205. doi: 10.1016/j.jdermsci.2007.07.003 17719747

[23] Mcgowan KM Coulombe PA. Onset of keratin 17 expression coincides with the definition of major epithelial lineages during skin development. J Cell Biol (1998) 143(2):469–86. doi: 10.1083/jcb.143.2.469 PMC2132846 9786956

[24] Yunusbaeva M Valiev R Bilalov F Sultanova Z Sharipova L Yunusbayev B. Psoriasis patients demonstrate hla-Cw*06:02 allele dosage-dependent t cell proliferation when treated with hair follicle-derived keratin 17 protein. Sci Rep (2018) 8(1):6098. doi: 10.1038/s41598-018-24491-z 29666398 PMC5904118

[25] Steiner JE Cottrell CE Streicher JL Jensen JN King DM Burrows PE. Scarring in patients with Pik3ca-related overgrowth syndromes. JAMA Dermatol (2018) 154(4):452–5. doi: 10.1001/jamadermatol.2017.6189 PMC5876829 29516089

[26] Xu F Na L Li Y Chen L. Roles of the Pi3k/Akt/Mtor signalling pathways in neurodegenerative diseases and tumours. Cell Bioscience (2020) 10(1):54. doi: 10.1186/S13578-021-00667-5 32266056 PMC7110906

[27] Hua L Zhou Y Hou C Chen J Wang Y Zhang S. Shexiang baoxin pills inhibited proliferation and migration of human coronary artery smooth muscle cells *Via* Pi3k/Akt/Mtor pathway. Front Cardiovasc Med (2021) 8:700630. doi: 10.3389/fcvm.2021.700630 34513945 PMC8425485

[28] Wang X Angelis N Thein SL. Myb - a regulatory factor in hematopoiesis. Gene (2018) 665:6–17. doi: 10.1016/j.gene.2018.04.065 29704633 PMC10764194

[29] Cicirò Y Sala A. Myb oncoproteins: Emerging players and potential therapeutic targets in human cancer. Oncogenesis (2021) 10(2):19. doi: 10.1038/s41389-021-00309-y 33637673 PMC7910556

[30] Jin Y Zhu H Cai W Fan X Wang Y Niu Y. B-myb is up-regulated and promotes cell growth and motility in non-small cell lung cancer. Int J Mol Sci (2017) 18(6):860. doi: 10.3390/ijms18060860 28555007 PMC5485926

[31] Fan X Wang Y Jiang T Cai W Jin Y Niu Y. B-myb mediates proliferation and migration of non-Small-Cell lung cancer *Via* suppressing Igfbp3. Int J Mol Sci (2018) 19(5):1479. doi: 10.3390/ijms19051479 29772705 PMC5983693

[32] Zhang H Jiang S Guo L Li X. Microrna-1258, regulated by c-myb, inhibits growth and epithelial-To-Mesenchymal transition phenotype *Via* targeting Sp1 in oral squamous cell carcinoma. J Cell Mol Med (2019) 23(4):2813–21. doi: 10.1111/jcmm.14189 PMC6433684 30734471

[33] Lee YH Kim HS Kim JS Yu MK Cho SD Jrno JG. C-myb regulates autophagy for pulp vitality in glucose oxidative stress. J Dent Res (2016) 95(4):430–8. doi: 10.1177/0022034515622139 26661713

[34] Bu C Xu L Han Y Wang M Wang X Liu W. C-myb protects cochlear hair cells from cisplatin-induced damage *Via* the Pi3k/Akt signaling pathway. Cell Death Discov (2022) 8(1):78. doi: 10.1038/s41420-022-00879-9 35210433 PMC8873213

[35] Zubair H Patel GK Khan MA Azim S Zubir A Singh S. Proteomic analysis of myb-regulated secretome identifies functional pathways and biomarkers: Potential pathobiological and clinical implications. J Proteome Res (2020) 19(2):794–804. doi: 10.1021/acs.jproteome.9b00641 31928012 PMC7700759

[36] Takeo M Lee W Ito M. Wound healing and skin regeneration. Cold Spring Harbor Perspect Med (2015) 5(1):A023267. doi: 10.1101/cshperspect.a023267 PMC4292081 25561722

[37] Wilkinson HN Hardman MJ. Wound healing: Cellular mechanisms and pathological outcomes. Open Biol (2020) 10(9):200223. doi: 10.1098/rsob.200223 32993416 PMC7536089

[38] Singer AJ Clark RA. Cutaneous wound healing. N Engl J Med (1999) 341(10):738–46. doi: 10.1056/NEJM199909023411006 10471461

[39] Freedberg IM Tomic-Canic M Komine M Blumenberg M. Keratins and the keratinocyte activation cycle. J Invest Dermatol (2001) 116(5):633–40. doi: 10.1046/j.1523-1747.2001.01327.x 11348449

[40] Zhang X Yin M Zhang LJ. Keratin 6, 16 and 17-critical barrier alarmin molecules in skin wounds and psoriasis. Cells (2019) 8(8):807. doi: 10.3390/cells8080807 31374826 PMC6721482

[41] Vo PT Choi SS Park HR Lee A Jeong SH Choi Y. Gene signatures associated with barrier dysfunction and infection in oral lichen planus identified by analysis of transcriptomic data. PloS One (2021) 16(9):E0257356. doi: 10.1371/journal.pone.0257356 34506598 PMC8432868

[42] Charles CA Tomic-Canic M Vincek V Nassirri M Stojadinovic O Eaglstein WH. A gene signature of nonhealing venous ulcers: Potential diagnostic markers. J Am Acad Dermatol (2008) 59(5):758–71. doi: 10.1016/j.jaad.2008.07.018 PMC4994807 18718692

[43] Batzer AT Marsh C Kirsner RS. The use of keratin-based wound products on refractory wounds. Int Wound J (2016) 13(1):110–5. doi: 10.1111/iwj.12245 PMC7949970 24580740

[44] Pechter PM Gil J Valdes J. Keratin dressings speed epithelialization of deep partial-thickness wounds. Wound Repair Regener (2012) 20(2):236–42. doi: 10.1111/j.1524-475X.2012.00768.x 22332782

[45] Adair HM. Epidermal repair in chronic venous ulcers. Br J Surg (1977) 64(11):800–4. doi: 10.1002/Bjs.1800641113 588976

[46] Falanga V. Wound healing and its impairment in the diabetic foot. Lancet (2005) 366(9498):1736–43. doi: 10.1016/S0140-6736(05)67700-8 16291068

[47] Menke NB Ward KR Witten TM. Impaired wound healing. Clinics Dermatol (2007) 25(1):19–25. doi: 10.1016/j.clindermatol.2006.12.005 17276197

[48] Stojadinovic O Pastar I Vukelic S Mahoney MG Brennan D Krzyzanowska A. Deregulation of keratinocyte differentiation and activation: A hallmark of venous ulcers. J Cell Mol Med (2008) 12(6b):2675–90. doi: 10.1111/j.1582-4934.2008.00321.x PMC3828883 18373736

[49] Grinnell F Ho CH Wysocki A. Degradation of fibronectin and vitronectin in chronic wound fluid: Analysis by cell blotting, immunoblotting, and cell adhesion assays. J Invest Dermatol (1992) 98(4):410–6. doi: 10.1111/1523-1747.ep12499839 1372338

[50] Reiss MJ Han YP Garcia E Goldberg M Yu H Garner WL. Matrix metalloproteinase-9 delays wound healing in a murine wound model. Surgery (2010) 147(2):295–302. doi: 10.1016/j.surg.2009.10.016 20004432 PMC2813947

[51] Stojadinovic O Pastar I Nusbaum AG Vukelic S Krzyzanwska A Tomic CM. Deregulation of epidermal stem cell niche contributes to pathogenesis of nonhealing venous ulcers. Wound Repair Regener (2014) 22(2):220–7. doi: 10.1111/wrr.12142 PMC4329920 24635172

[52] Sawaya AP Stone RC Brooks SR Pastar I Jozic I Hasneen K. Deregulated immune cell recruitment orchestrated by Foxm1 impairs human diabetic wound healing. Nat Commun (2020) 11(1):4678. doi: 10.1038/s41467-020-18276-0 32938916 PMC7495445

